# “How Do They Even Know They Love?” The Image of Polyamory in Polish Expert Discourse

**DOI:** 10.1007/s10508-020-01787-8

**Published:** 2020-07-28

**Authors:** Katarzyna Grunt-Mejer, Weronika Chańska

**Affiliations:** 1 The Faculty of Psychology and Law in Poznań, SWPS University of Social Sciences and Humanities, 61-719 Poznan, Poland; 2 Faculty of Health Sciences, Jagiellonian University Medical College, Kraków, Poland

**Keywords:** Polyamory, Psychotherapists, Media discourse, Mononormativity, Poland

## Abstract

The article presents the results of a thematic analysis of statements about polyamory made in the media by Polish psychology and sexology experts. The analysis was conducted on the basis of 20 pieces of material released in the Polish national press, radio, and television between July 2012 and October 2018. The results show that most of the analyzed experts approach polyamory with suspicion. In most cases, the decision to be in a polyamorous relationship is assessed very negatively, and in the eyes of the therapists it is evidence of psychological defects in people who make such attempts or it is seen as a harbinger of unfavorable outcomes for the relationship. This negative psychological evaluation is often accompanied by a strong moral assessment and a clear willingness to discourage society from this relationship model. The results show that representatives of psychology and medical sciences in the Polish media support and legitimize the social and moral order that promotes mono-normativity. The true reasons for the aforementioned negative assessment are hidden behind a veil of scientific objectivity.

## Introduction

The term “polyamory” is used to denote “the practice of, belief in, or willingness to engage in multiple romantic and/or sexual relationships with the consent of everyone involved” (Rubel & Bogaert, 2015). Although the term itself is relatively new, the concept and its underlying ideas have been known for decades. Group marriages in the 1970s (Constantine & Constantine, 1973; Edwards & Stinnett, 1974), among others, were built on similar premises. Their distinguishing feature was, as is the case with polyamory, the partners’ consent to romantic and sexual relations with other people as well as the possibility of maintaining a common household and collective parenting. The last two decades have seen increased interest in polyamory from the scientific community. Research into polyamory is conducted as part of exploration of a wider spectrum of phenomena collectively referred to as consensual nonmonogamy (CNM). The term is used to denote relationships in which the partners consent to having sexual, romantic, or intimate relationships with more than one person (see Barker & Langdridge, 2010). Research shows that 1 out of 5 Americans and Canadians has made attempts at or had experiences of creating CNM relationships (Fairbrother, Hart, & Fairbrother., 2019; Haupert, Gesselman, Moors, Fisher, & Garcia, 2017). There is also ongoing investigation whose aim is to establish the relationship satisfaction level and psychological profile of people who decide to engage in CNM. Research results provide evidence that people who choose the monogamous relationship style and those who prefer consensually nonmonogamous styles have a lot in common. The groups score similarly on personality traits scales (Twichell, 1974; Watson, 1981), and on mental health indicators such as alienation, depression, anxieties and phobias, life satisfaction, self-esteem, neuroticism, paranoid ideations, psychoticism and hopelessness (Buunk, 1980; Murstein, Case, & Gunn, 1985; Parsons, Starks, DuBois, Grov, & Golub, 2013; Wagner, Remien, & Dieguez, 2000). The monogamous and nonmonogamous groups also show comparable levels of relationship satisfaction, commitment and sexual satisfaction (Blasband & Peplau, 1985; Buunk, 1980; Hoff, Beougher, Chakravarty, Darbes, & Neilands, 2010; Kurdek & Schmitt, 1986; LaSala, 2004; Mitchell, Bartholomew, & Cobb, 2014; Morrison, Beaulieu, Brockman, & Beaglaoich., 2013; Murstein et al., 1985; Ramirez & Brown, 2010; Rubin, 1982; Rubin & Adams, 1986; Wagner et al., 2000)

There are also studies that point to certain differences between people who enter CNM relationships and those who prefer sexually and emotionally exclusive relationship models. Studies claim that people who choose the latter relationship styles have more positive childhood memories (Gilmartin, 1974) and maintain closer relations with their families (Gilmartin, 1974; Twichell, 1974) than people who have nonmonogamous relationships. Some research also indicates that people in CNM relationships seek psychological help more often (Murstein et al., 1985; Smith & Smith, 1970). At the same time, other studies have shown that people in CNM relationships (mainly swingers) report higher levels of excitement and lower levels of boredom (Bergstrand & Williams, 2000; Gilmartin, 1974; Murstein et al., 1985). They are also characterized by stronger social bonds and lower levels of anomie as compared to monogamous people (Gilmartin, 1974). Research also indicates a stronger ability to cope with jealousy in people who engage in CNM relationships (Jenks, 1985).

Comparisons between monoamorous and polyamorous people (in the narrow sense of the term, i.e. excluding other types of consensual nonmonogamy) show results similar to those conducted on broadly defined groups of people who maintain CNM relationships. Polyamorous people exhibit higher relationship intimacy levels than monoamorous people (Morrison et al., 2013). They also declare high levels of need fulfillment and satisfaction in their plural relationships (Mitchell et al., 2014).

The studies quoted demonstrate that people who choose consensual nonmonogamy, including polyamory, resemble those who choose monogamous relationships in many ways that are relevant from a psychological standpoint. The research also shows that the form of the relationship (open or closed, allowing or disallowing sexual and emotional relations with people outside the couple) do not predetermine relationship satisfaction level and do not impact the degree of sexual satisfaction.

### Existing Research on Attitudes Toward Consensual Nonmonogamy

The conclusions above are not reflected in the social perception of people who have CNM relationships: they are judged more negatively than partners in monogamous relationships. The relationships themselves (e.g. to what degree they satisfy the needs of the partners) are also assessed more unfavorably.

Compared to the various types of CNM relationships (polyamory, swinging or/and open relationships depending on the study), the effect of strong positive valorization of monogamy was visible in all studies on lay people’s attitudes towards consensually nonmonogamous relationships. Numerous merits are attributed to monogamy, from STI prevention (Balzarini, Shumlich, Kohut, & Campbell, 2018; Conley, Moors, Matsick, & Ziegler, 2013; Hutzler, Giuliano, Herselman, & Johnson, 2016; Moors, Matsick, Ziegler, Rubin, & Conley, 2013), through protecting the relationship from ending and promotion of its longevity (Burleigh, Rubel, & Meegan, 2017; Edwards & Stinnett, 1974; Grunt-Mejer & Łyś, 2019), to increased relationship and sexual satisfaction (Burleigh et al., 2017; Cohen, 2016; Conley et al., 2013; Grunt-Mejer & Campbell, 2016; Grunt-Mejer & Łyś, 2019; Moors et al., 2013).

Moreover, people who maintain CNM relationships were on many levels assessed more negatively than people who choose monogamous relationships. The former have been attributed with higher levels of promiscuity (Balzarini et al., 2018; Hutzler et al., 2016), lower morality (e.g. trustworthiness, or orientation towards other), lower cognitive abilities (e.g. intelligence, coping in difficult situations, Burleigh et al., 2017; Conley et al., 2013; Grunt-Mejer & Campbell, 2016; Grunt-Mejer & Łyś, 2019; Hutzler et al., 2016; Thompson, Bagley, & Moore, 2018), and increased loneliness (Moors et al., 2013). These results are repeatable and consistent regardless of the methods used (explicit measures of attitudes, implicit measures (IAT) and non-direct measures of attitudes, such as desired social distance ratings and measures of dehumanization through the attribution of primary and secondary emotions).

However, the assessment of different types of CNM is nuanced. Usually swingers are perceived more negatively or polyamorous people are perceived more positively than people in other CNMs (Balzarini et al., 2018; Cohen, 2016; Grunt-Mejer & Campbell, 2016; Matsick, Conley, Ziegler, Moors, & Rubin, 2014). Additionally, although people who engage in monogamous relationships are perceived more favorably, the general ratings of CNM relationships are above mid-points and are definitely higher than ratings of unfaithfulness (Burris, 2014; Edwards & Stinnett, 1974; Grunt-Mejer & Campbell, 2016; Grunt-Mejer & Łyś, 2019). Also, not all traits of people engaging in CNM are perceived as worse than those of monogamous people: polyamorous people are sometimes assessed as better at communication, more extravert, more attractive, and better at coping with jealousy (Grunt-Mejer & Campbell, 2016; Hutzler et al., 2016; Johnson, Giuliano, Herselman, & Hutzler, 2015).

The aforementioned research therefore demonstrates that polyamory is a favored form of CNM and is more positively evaluated by society than other forms of CNM. The contradistinction between swinging, open relationships and polyamory could indicate the role of love in the formation of judgments (Cohen, 2016; Grunt-Mejer & Campbell, 2016; Matsick et al., 2014). Although still transgressive, polyamory may gain more acceptance in people’s eyes due to “its up-front endorsement of love” and by contesting the more sex-driven forms of relationships (Klesse, 2011, p.5).

Similar results can be observed in studies on mental health professionals’ attitudes towards people who engage in CNMs. Older research shows that psychologists often attribute numerous unfavorable traits to general CNM, including traits indicative of mental disorders (Constantine, Constantine, & Edelman., 1972; Hymer & Rubin, 1982; Knapp, 1975). The subset most negatively assessed is swingers, who in the perception of psychotherapists exhibit more emotional issues than people who cheat on their partners (Hymer & Rubin, 1982; Knapp, 1975).

Some contemporary research suggests that psychotherapists have a positive attitude to polyamory overall (Stavinoha, 2017). Most people who engage in CNM who have sought psychotherapeutic help also report positive experiences as pertains to the therapist’s attitude to the client’s unconventional relationship style (Schechinger, Sakaluk, & Moors et al., 2018). Nevertheless, respondents listed some negative experiences with therapists: their disbelief in a polyamorous client’s attachment to multiple partners; their denying or minimizing of the value of clients’ relationships; their attribution of the cause of the presented problem to nonmonogamy; the fact that they sometimes manipulate or apply pressure in order to restore clients to a monogamous lifestyle; and a judgmental and shaming attitude towards CNM (Henrich & Trawinski, 2016; Schechinger et al., 2018).

There are also data indicating that therapists assess the relationship satisfaction of polyamorous people and their level of morality as lower than monogamists; they also believe that persons who choose polyamory do worse in life. In the case of people in CNM relationships who seek professional help, the source of the reported problem is more often attributed by therapists to the type of relationship the clients form. In turn, problems reported by monogamists are rarely linked to the dynamics and quality of their intimate relationships (Grunt-Mejer & Łyś, 2019).

### Aims of the Current Study

These studies indicate a significant bias in the attitudes of some therapists that is conditioned by mono-normative culture. However, the fact that therapeutic work is not the only area in which lay people are influenced by psychology should be taken into account. Psychologists influence people not only in therapy but also by presenting their opinions and expertise in popular media. Thus, we should ask what kind of messages are sent by professionals in the public discourse on polyamory that consequently shape its image in the mind of the lay person.

Historical research shows that there is a correlation between psychological findings and social norms. On the one hand, psychologists are the children of their own time: they accept as obvious and indisputable the messages of the culture in which they live and they create the theories and methods of their research on this basis. On the other hand, psychology, by virtue of the authority it derives from being a science, can both legitimize and disprove popular beliefs about human functioning. Thus, it can both perpetuate the existing status quo or critically analyze the social order, thereby fostering social change (Prilleltensky, 1989, 1994; Sarason, 1981).

Mental health experts are seen by members of the public as having the power to formulate specific normative opinions (good or bad, acceptable or socially undesirable) on the phenomena on which they comment (Rose, 1990). These opinions then become a source of the individual assessments and attitudes of the expert audience. Social reluctance to new, unusual or divergent phenomena is thus supported by the authority of science. This in turn may facilitate and legitimize the social ostracism of people who behave differently than is accepted. Conversely, the authority of science may initiate the process of changing existing social attitudes and introduce the spirit of tolerance and respect for diversity.

The aim of our study was to examine how Polish mental health experts use the opportunity to shape public opinion when they have a chance to reach a wide audience. We were interested in whether people who are considered to be an authority in psychology would use their expertise to present the current research findings or adhere to the social stereotypes and bias against polyamorous relationships.

## Method

### Sources

The source of our data was statements about polyamory made in media interviews by Polish experts, psychologists and sexologists. The analysis encompassed all interviews the authors were aware of that were released in national press, radio and television between July 2012 (when the very first article on polyamory appeared in the mainstream media) and October 2018. The analysis included a total of sixteen press articles, two TV programs and two radio broadcasts (all the sources are listed in “Appendix 1”; the numbers given in the text refer to the numbers in the source list). The analyzed statements were made by twenty-one experts, nineteen of whom are practicing psychotherapists and two of whom are well-known professors specializing in social psychology. They are persons who are commonly recognized and perceived as role models and who are employees at institutions that provide professional training to future psychologists and therapists.

### Analysis

We used the guidelines defined in the work of Braun and Clarke (2013) for thematic analysis. Although our analysis was guided by the main goal of the study, i.e. the presentation of experts’ opinions on polyamory, our approach to the data was not guided by existing theories and the themes were identified in an inductive way. The analysis was also constructionist in its attempt to identify not only what kind of associations experts have in relation to polyamory, but also what kind of deeper assumptions about relationship ideals the experts are informed by and what type of social order they (not necessarily consciously) prescribe.

The source material was analyzed multiple times in order to extract the most recurring themes. The identification of themes was conducted independently by the two authors. Next, the two analyses were contrasted and on this basis the final list of the most frequently recurring themes was established. In a few cases the analysis was supplemented with themes that appeared in single interviews but allowed a more thorough understanding and explanation of the main subject of the analysis.

Three major themes (the image of love, the profile of a polyamorous person, and the consequences of forming polyamorous relationships) and 23 minor themes were identified.

## Results

### The Image of Love

Experts’ ideas of how good love and successful relationships should look form the background for talking about polyamory. Polyamorous people are perceived through the prism of their alleged personal qualities and their willingness to create loving relationships that fulfil the assumed relationship ideal.

#### Humans Are Naturally Monogamous

The vast majority of the studied experts hold the opinion that people are naturally monogamous or serially monogamous. To strengthen their claim, they use arguments grounded in biology:

It’s motivated by biology because even if we don’t want to admit it, we are, to some extent, animals, and the vast majority of us belong to the three percent of species that are recognized in the natural world as monogamous. [12]

Sometimes they also refer to statistics showing that the vast majority of people have: “So why do as many as 92 percent of us have typical, monogamous preferences?” [12]

Some of the experts mention psychological theories that are said to prove humans’ need for monogamy, but these theories are never named explicitly. The experts only say that they exist or that most psychological theories state this.

There are, however, some statements which admit that it is possible to love a few people at the same time [3,4,11]:

Is it possible to love multiple people at once? It is. We do love our mother and father, our siblings, our husband, our children, and even the dog and the cat. We love our partners and our lovers at the same time. In various cultures there have always been relationship models where there was more than one partner. [3]

One of the experts mentions that love is a subjective emotion and if someone “feels that they love someone then psychology has nothing to do with establishing if this person is experiencing it inadequately” [11]. This expert, in contrast to the previously quoted specialists who questioned the possibility of loving a few people at the same time, clearly relinquishes the right to judge whether polyamorous love is more or less real than monoamorous love: “I know polygamists who have 2–3 wives and say that their love towards each of them is different. I can’t deny the fact that they do love” [11].

#### A True Relationship Is a Total Relationship

The experts maintain deep skepticism towards the possibility of making and effectively executing a decision to engage in an alternative lifestyle: “I’m not convinced it’s doable to just make a logical agreement like “listen, in case of anything, we’re just not going to be jealous” [19]. The examined experts also point to numerous human needs and desires that can only be satisfied in a monogamous relationship, such as “the natural thirst for intimacy” [8] and the desire to form a truly intimate, close relationship. A true relationship is based on sharing one’s whole life with a partner [4].

In a few instances, the experts share the notion of the total nature of intimate relationships by which the partners become one [12]. People who form such relationships understand the need for “a healthy dependency” [13] which thoroughly justifies the desire to keep the partner only to themselves: “Love means I long for my chosen one to be with me” [12].

#### The Myth of the Two Matching Halves

The vast majority of experts in our sample are convinced that true love is love for one person. To support this claim, one of the experts references the results of a public opinion survey in which most of the respondents said they were truly in love only once in their lifetime [12]. This opinion is sometimes enhanced by a reference to the myth of “the two matching halves.” It states that once we find the right person, it will become completely obvious to us that we do not need anyone else:

If two so-called matching halves meet–which happens rarely at first attempt–then faithfulness is not an issue. It’s biologically, psychologically and spiritually obvious. It doesn’t require a conversation, an agreement or a guard. It isn’t related to feeling restricted or to loss of freedom. In a relationship like that, no one would think of having intimate relations with anyone else. [14]

However, the experts say that when we are unable to create a perfect relationship like this, we can be tempted to seek other relationship models such as polyamory. But the search cannot make us happy and we will abandon it as naive as soon as we find the right person [14].

Finding “the other half”—as one expert puts it—makes them become “our passion” [15], and this in turn causes us to desire exclusive access to the person: “If we are deeply interested in another person, we won’t put up with them easily forming other relationships. We will impose a monogamous model on them” [11] or “If the other person becomes our passion, then sharing simply won’t work” [15].

#### Being the One Who is Chosen

In the eyes of the experts, a true love encompasses a sense of having been chosen, a sense of uniqueness, and this can be provided only by monogamy. Polyamory is just the opposite: “In an arrangement like that, I’m not unique and special, the most important one for my partner. The emotional cost of such an arrangement is tremendous” [4].

The studied experts state that the sense of uniqueness (being the only person to be chosen by the partner) is accompanied by high self-esteem: “Usually, being exclusive with someone makes us feel chosen by this person. This reinforces our value in our own eyes” [7].

Others additionally point to the issue of a partner’s faithfulness as a factor in one’s own self-esteem [15].

#### True Love is Other-Directed

The experts are of the opinion that to form a good relationship people must have certain characteristics. They must be other-directed and to some extent self-forgetful. Partners are supposed to take each other’s needs seriously, to communicate their own needs effectively and be prepared to forsake some of these needs in order for the partner’s needs to be satisfied [13].

In contrast, people who form polyamorous relationships are perceived by the experts as overly concerned with their own desires, needs, or interests: “Polyamory is a form of self-centeredness, a desire to satisfy one’s needs more than the other’s” [12]. Polyamorous persons are depicted as extremely self-seeking:

A relationship between people is not only outbursts of passion, excessive emotions, some game or an interplay of self-interest, but also the ability to actually respect the partner. And respect means treating them as having unique value. Not a sex object, not a social convenience, not a tool for self-gain. I wonder if these components of respect are at all significant in polyamorous relationships and whether it’s possible that they would be ever truly present. [9]

#### The Labor of Love

The experts believe that togetherness requires high levels of attention and effort on the part of the partners and that it is not possible for anyone to make such intense efforts with more than one person. As one of the experts puts it, “our mental space is very narrow and not many people fit into it” [15].

The desire to create open relationships is seen by the experts as evidence of someone’s “defectiveness”—their inability or unwillingness to take up the “labor of love.” The statements of people who claim that they cannot find their way in traditional, monogamous relationships are treated by the experts as proof of a lack of proper reflection and the ability to draw conclusions from previous failures. In such situations, experts recommend that clients investigate their past experiences more thoroughly and draw conclusions. Only this type of deep self-processing carries the hope of finding happiness, which, is some the experts’ opinions, is only possible in a monogamous relationship:

There’s always an answer to the question of “why isn’t it working for us” because it is ourselves that are creating the relationship. If we find it, our future relationships will be better–longer, deeper, more authentic–perhaps we’ll even find the one, forever and ever. [8]

#### Monogamy as a Moral Practice

The notion of commitment is strongly linked with the belief in the necessity and value of putting continuous effort into the relationship. It is correlated with taking on numerous commitments to the partner. One of the most significant of these is the commitment to be faithful (interpreted by the experts as excluding not only cheating, but also any sexual contact with other people undertaken with both partners’ consent): “In our part of the world, monogamy is a model chosen by the majority as a kind of noble obligation and a chance to practice the virtue of sexual restraint” [14].

A decision to engage in a parallel relationship, regardless of whether it is with the partner’s consent or not, is perceived by the experts as a form of moral collapse that is rooted in loss of self-control [9]. Constant fight and resisting temptation are, in turn, signs of “spiritual development” [8]. Practicing faithfulness as a kind of spiritual development exercise for many years carries the hope of forming the ideal relationship:

Also when we experience and perceive our relationship as mismatched, the decision to remain faithful is important and right as it lets us test ourselves and mature. Many young and immature people are incapable of faithfulness. Their unfaithfulness may not have anything to do with the choice of partner. Then, we experience the commitment to be faithful as an effortful exercise in inner discipline. That’s good. If we can last long enough, we will mature, the other person will mature and our relationship may turn out to be what we need and what we want to protect. [14]

This constant self-disciplining and cultivating the notion of sacrifice also bring hope in the stability of the relationship once both partners’ passion and strong feelings have faded [12].

At the same time, the experts do realize the fact that many people experience various kinds of “slipups.” These usually take the form of cheating. Coming off the virtuous rails in this way does not have to mean an end to the relationship. It should, however, become a motivator for thorough remedial work: “As long as both partners jointly take responsibility for one of them cheating, they can reform themselves and their relationship.” [14]

According to the experts, a relationship, as a two-person venture, requires joint action. When a crisis takes place, it can only be averted when both partners reinforce once again (and this time in a lasting way) their willingness to take on the commitment to remain faithful to each other [14]. In some cases it is better not to expect in return a specific kind of reward in the form of affectionate or effusive gestures on the part of the partner. Those who can remain faithful by the sheer strength of their commitment and do not expect too much from life are sometimes happier. As one of the experts puts it:

Relationships that can survive at such a modestly laid table are due the utmost respect. […] Wise people are happy not only because they don’t long to possess things, but also because they aren’t emotionally greedy. [14]

#### Relationships are Governed by Rules

Among the expert opinions, apart from the image of the ideal match of people who are destined for each other, we can also find those that define a relationship as a form of contract. Some of the experts put it in highly conservative terms: “Usually, a relationship is two people who trade services: men provide protection, women provide faithfulness”. [13] Other experts allow some freedom in how relationships are shaped by the partners, but the value and necessity of clear rules is always emphasized: “Every relationship is a kind of contract between people, no matter if it’s open or closed. In order to feel emotionally secure, it’s necessary to establish clear boundaries and a precise agreement on what is allowed and what isn’t”. [3]

The rules (usually understood by the experts as a series of restrictions) are meant to build a sense of security for the people who form the relationship: “Our sense of security has two main sources: order and predictability in the environment we live in and our inner sense of agency.” [9]

From this perspective, monogamous relationships, even in their imperfect, temporary form, seem much more desirable than any other form of relationship: “Serial monogamy is more orderly. The operating rules are clearer and more predictable. It’s an arrangement in which the sense of certainty and security is higher than in collectives.” [9]

Only one of the analyzed statements contains the belief that norms can be created independently by the partners. As long as they are established together by everyone involved and with everyone’s consent, they don’t hurt anyone’s interest and they can still help develop a sense of trust and security: “If each participant of such a relationship is in it voluntarily and fully consciously, the relationship satisfies partnership norms.” [7]

#### One Can Learn from Polyamorous Experience

Among the experts’ opinions a few can be found in which they also attempt to see some value in polyamorous relationships. First of all, they are tolerated to a certain extent as long as the people who engage in them are young and inexperienced. In that case, some of these experts perceive these experiments as a form of training in acquiring the skills that are necessary to create “authentic relationships” (i.e. monogamous ones):

Leaving aside the moral dimension, a polyamorous arrangement can be great social and relationship training. It helps practice some vital emotional components. It teaches communication and the ability to tell each other difficult things. Coping with emotions, and especially (which is highly important!) with jealousy. It helps understand that sex and relationships are different with different people, so it’s easier to accept otherness and partners’ flaws. […] An experience like this can help in building relationships in the future. [3]

People who maintain polyamorous relationships can also, to some extent, be an inspiration to monogamous couples in terms of more equal sharing of responsibilities [19].

The subject of working through jealousy returns in a few experts’ statements. This time, the techniques used by people who practice polyamory might help persons who have been cheated on by their partners:

As I was reading “The Ethical Slut,” I instantly had the thought that I would recommend it to women who are being cheated on. For me, it’s valuable because it teaches you how not to suffer because of jealousy. It teaches how to cope with it–how to explain some things to oneself rationally in order not to generate emotions that destroy us. [15]

This comes down to realizing our own value, regardless of whether we are the only person our partner has established a close relationship with. The experiences of polyamorous people can teach us “that we are valuable in ourselves, for ourselves, and that we should never judge ourselves on the basis of someone choosing another person” [15].

Therefore some techniques used in polyamorous relationships can be a salve for the wounds inflicted by a cheating partner in a monogamous (in principle) relationship.

#### Interpretative Analysis of the Experts’ Image of Love

The experts see monogamy as a biological trait of the human species and claim that most societies are built on monogamous relationships. Thus, they try to convey to the public that what is biologically underpinned and practiced by most people is also desirable and morally correct by necessity. In contrast to these normative statements, fewer voices indicate the possibility of loving several people at once. The experts who raise them refer to specific cases, not theories. They do not attempt to create an all-encompassing theory of love but point to the subjectivity of this emotion and the need to trust individual human experience. As we will show in the following themes, the same experts who allow the possibility of polyamorous love also have less inclinations to see polyamorous people as psychologically disturbed and to predict the disastrous consequences of polyamorous relationships.

Some of the experts see a good relationship as a “total” relationship, i.e. one in which the partners share all their experiences and satisfy all of each other’s needs. Such a relationship is made possible by finding the right person, your “other half.” Dissatisfaction with a relationship is explained by the experts either as a result of the wrong choice of partner (not finding the “right one”) or as a result of the lack of proper level of commitment or work put in the relationship. On this occasion, the experts’ statements show a characteristic oscillation between the vision of the kind of love that is natural and given at the moment of finding the right person, and the kind of love which requires constant work and moral improvement of the partners. It seems that the experts are not aware of the inconsistencies of their visions of love and use one or the other rather freely to criticize the alleged shortcomings of polyamorous relationships.

The “labor of love,” which appears in the statements of the experts as an important element of a happy relationship, places many demands on the partners: the ability to identify their own needs and communicate them effectively to their partner and to keep their own needs and desires restricted in the name of their partner’s best interest or the more abstractly defined “good relationship.” Moreover, the “labor of love” requires a great deal of time, effort and attention. This belief is at the root of the argument that it is impossible to create a happy relationship with more than one person. According to the studied experts, it is not possible for anyone to devote so much time and attention to more than one partner. This assumption fits in the minds of the experts with the conviction that people who form polyamorous relationships are necessarily more selfish and unwilling to self-limit and give up some of their own needs for the benefit of their partner, and therefore unable to do the “labor of love.” Thus, the relationships created by them cannot be happy.

The “work of love” is founded on a series of voluntary commitments. The most important of these is the commitment to be faithful to your partner. In this context, faithfulness gains exceptional value as keeping it is a way of practicing inner discipline. The decision to make the effort to remain faithful is motivated not only by respect and care for the partner, but also by cultivating one’s own perfection and exercising virtue. Many of the experts more or less directly express the belief that an authentic intimate relationship between two people can only be realized through each person making an unconditional commitment to remaining in the relationship. It is a form of an original act that governs all later commitments. It relies not so much on choosing a partner based on their traits (as then it would no longer be binding once the partner’s traits change), but rather it is a sort of autonomous decision (which is binding because of the very fact it was made, not what motivated it). Once the partners decide to form a monogamous relationship, their whole future life is supposed to be organized around maintaining this decision, reinforcing the validity of their choice, and practicing behaving accordingly. The partner’s traits or behavior are not significant (although finding the perfect “other half” makes it easier to meet this commitment). A crucial role is played by each partner’s own perseverance, consistency and ability to coerce themselves into certain actions imposed by their own will. Love, according to many of the experts, is realized through self-discipline.

The experts realize that infidelities happen in many relationships. However, the mere fact of cheating is not enough to end the relationship. Rather, it should serve as an encouragement to take a critical look at the past and make an effort to repair the relationship. Improvement can only be achieved by restraining one’s sexual appetites and putting the will to be faithful above any temptation. This kind of “spiritual discipline” can improve the quality of the relationship, but even if this does not happen, the very fact of this moral victory over temptations will, according to many of the experts, be rewarding.

It is also worth noticing that the experts quite consistently use the term “cheating” to refer to any form of relationship which does not presuppose the monogamous model. They do not distinguish between situations in which a third person enters the relationship secretly and without one partner’s consent and those in which finding new partners takes place with everyone’s knowledge and with the consent of everyone involved. Every form of departure from the monogamous model is automatically classified as a form of “cheating.” As we will show later in the analysis, some of the experts are convinced that the term “polyamory” is only used to cover old and common human sins, therefore a polyamorous person does not differ in any particular way from a person with questionable sexual ethics.

In the experts’ statements about what a relationship is, one can also find the conviction that it is a kind of contract. Drawing on the contract metaphor is meant to emphasize the fact that monogamous relationships follow certain long-known and never-changing rules. The existence of these rules provides the partner with a sense of certainty and predictability in relation to the other partner’s behavior. Polyamorous relationships appear to be completely formless and unpredictable in this context. The experts are therefore surprised that anyone can find them attractive.

Only a few people from our analyzed group try to see some good sides of polyamorous relationships. They are seen as short affairs that can be treated leniently if they concern young people: in this case they can be translated as a kind of “transition phase,” a process of maturing that leads to the creation of a “true,” i.e., monogamous, relationship. Being in a polyamorous relationship may contribute to the improvement of the communication skills of the partners and teach them a more equal division of duties. It is also good training in how to deal with jealousy.

### The Profile of a Polyamorous Person

One of the most recurring themes in the analyzed material is the traits attributed to polyamorous people. The experts devote most attention to this issue, attempting to create profiles of people who are interested in polyamory.

#### Fear of Intimacy and Commitment

Polyamorous people, according to the experts, “fear deeper relationships and loneliness or abandonment” [7], “can’t form intimate bonds,” and “don’t understand what healthy dependency is, how much happiness can be gained from closeness with another person—the faithfulness, intimacy and friendship which manifest with time in long-term relationships” [13]. Therefore, polyamory is “a form of escape from commitment, from responsibility. It’s an exceptionally attractive deal for people who have a problem with intimacy” [13] and as such is juxtaposed with a committed relationship: “I would say the opposite of polyamory is not marriage, it’s a relationship based on tremendous commitment” [15]. Consequently, a relationship characterized by commitment and responsibility would, according to the experts, lead to a sense of “seizure, appropriation and lack of space” [13] among polyamorous people.

One expert assumes that persons who practice polyamory are aware of their traits, but as they do not wish to address them they choose a label which is more convenient for their lifestyle, allowing them to be free of responsibility:

I think the people who fall under the “polyamory” label are people who have previously been uncomfortable living with another label: the commitment-phobe, a person who is afraid of true engagement, a person with a deep fear of loneliness, a person in an open relationship, or finally, a sexaholic. To me, these are concealed ailments, but when attractively labeled they start to be accepted. A person who is afraid of commitment knows they have a problem and should do something with the source of this problem. A polyamorist, thanks to the label, doesn’t have to do anything. [8]

To support the thesis of the need for intimacy and involvement in a good relationship, some of the studied experts refer to Sternberg’s triangular theory of love [9,12] but present it in such a way that the reader gets the impression that these components can only occur in monogamous relationships.

If the experts do give reasons for why some people have trouble with intimacy, closeness and commitment, they point to a difficult childhood: “It’s a profound problem rooted deep in childhood experiences. (…) In the language of psychology, this means an “insecure attachment style.” With time, the intimacy becomes threatening as the hormone levels decline” [13].

#### Self-Centeredness, Narcissism, and Discomfort Avoidance

The traits that are particularly frequently attributed to polyamorous persons are self-centeredness, narcissism and taking the easy way out. The experts assume that in the case of creating relationships that include more than two people, seeking new partners is self-serving and aims to “satisfy one’s own needs more than the other’s” [12]. This attitude, posited by the experts, is called “extremely self-seeking” [15], and in this case love only serves to justify the fact that one pulls from their partners whatever they need [15].

Consequently, polyamorous relationships do not require effort or sacrifice, commitment or giving anything [1]—people who practice polyamory are described as “taking the easy way out” [1] and pursuing personal interests [9]:”Being drawn to these kinds of relationships can be attributed to people who prefer a life of taking rather than giving, and for whom sacrifice is not a life motto” [13].

#### Emotional Maturity

The experts attribute two contradictory traits to polyamorous people: maturity or a lack thereof. This discrepancy can be explained by the experts by diverse understandings of the concept of maturity. Sometimes it is interpreted as the ability to recognize what we have:”Patients come in because they fail to see certain things, they don’t appreciate their partner, so when this person matures, the partner becomes truly enough” [5].

In another case, maturity means being accountable for partners’ feelings and caring for them:

We sexologists don’t discourage people from polyamorous relationships. But we do advise to choose them consciously and responsibly, which means remembering the “old” partners, who need attention, are flesh and blood, have feelings and can be hurt. All of this requires a high level of maturity [16].

Some, in turn, suggest that choosing a polyamorous lifestyle can be evidence of either an escape from growing up or an expression of maturity and courage in exploring human romantic relationships [14], In this case, the key factor in determining whether a person is mature is their ability to awaken in themselves the willingness to share their partner [14].

#### Problematic Sexual Standards

The experts devote a lot of attention to sexual matters when talking about polyamory. The subject of polyamory is often listed in one breath with the subjects of cheating, swinging, casual sex or ‘friends with benefits’. For some “it’s not the issue of love but the issue of sex that is more crucial in the phenomenon of polyamory” [15]. For others, it is sexaholism or having high sexual needs that are the main motivators for seeking new partners in polyamory [8,14].

Polyamory is particularly frequently perceived as a type of behavior that is immoral and a better-sounding cover-up for sexual exploration, which itself is referred to in negative terms such as debauchery, licentiousness, promiscuity, etc. [12].

Using the polyamorous label, according to the experts, also serves to veil dishonesty [11] or to justify having sexual contact with people outside the relationship [17].

Polyamory then appears to be a cover-up for problematic sexual standards:

[P]olyamory seems to me […] a deft ideology, usually justifying a deficiency in loyalty. If someone says that they are having intimate love relations with a few people, they will be perceived as quite an immoral person. If they say they’re the prophet of a higher form of love, then they become an advocate for a new (better?) morality. [9]

Only rarely are there voices that attempt to deconstruct the myth of the sexual insatiability, debauchery and promiscuity of polyamorous people [4]. Two experts [4,2] explicitly point out that people who seek solely to satisfy their excessive sexual needs will quickly be excluded from the polyamorous community: “Sometimes, people who are sex addicts or focused only on sex attempt to enter the polyamorous community. This causes them to be quickly rejected by the group” [2].

#### Elitists

According to the experts, resources are needed to maintain a multi-person relationship. As a result, people who engage in polyamory are well-off, urban, with a lot of spare time and a high social standing, which in turn is related to the fact that “polyamory is not an economical form of relationship” [13]:

If one is to treat the descriptions of this phenomenon seriously, then something quite interesting comes up (please excuse the triviality): polyamory requires sizeable economic resources and a lot of free time. Who has both? Who has either? Mainly well-off freelancers, e.g. artists. Also people with high social status. I’d say, not without a hint of mockery, that it’s a niche ideology, or even a parlor ideology. [9]

One expert infers social uselessness from the aforementioned resources. According to him this kind of behavior is particularly distinctive for “the leisure class” [12].

#### Manipulated

The experts often assume that one of the partners in a relationship was persuaded by the other to try a non-traditional relationship model and therefore the consent was only ostensible [4,12,13,14]. One expert explains:

The other half agrees because they’re in love, because they’re afraid they’ll be abandoned. It would be more honest if the one who is hungry for experimentation left and lived alone if they can’t do without polyamory and they feel that their current partner is agreeing to this despite themselves. [14]

The experts are also convinced that such partners “grin and bear it in order not to lose their partner, not to break up the family, not to turn the children’s lives upside down, wait it out until it ‘goes away’ and so on” [12], but they themselves remain “dissatisfied because they are not acting in accordance with their own values” [13].

Some assume that people who suggest a change of the relationship model from a monogamous to a polyamorous one already have an eye on someone else [17].

In other words, the experts often suppose that even if both partners expressed their willingness to try a new relationship model, then in the near future one of them will be at a disadvantage anyway because they will cease to be the object of love. In a few statements, it is either directly expressed or suggested that the partner who is put under pressure is the woman. Some even refer to evolutionary theories in order to justify men’s alleged higher interest in open relationships [12,18].

The assumption that one of the partners is not consenting authentically is sometimes accompanied by attempts at some possible explanations for why a person may consent to something that they do not really want:

Those who haven’t been loved whole-heartedly—especially if they didn’t feel loved whole-heartedly by the parent of the opposite sex—will unconsciously seek partners who are incapable of committing with their hearts (…) The same mechanism may be at the root of a partner who seemingly consents to polyamory. [14]

We also noted a statement which allowed a situation in which a partner may consent to polyamory despite their own reluctance. But in this case it is not treated as a case of giving into pressure, but rather as a testament to “a strong self-identity and our partner not neglecting us, and us feeling important enough to them.” [15]

#### Reasons for Entering a Polyamorous Relationship

Apart from the aforementioned reasons for an interest in polyamory due to personality, the experts mention burnout, weariness, curiosity (including sexual curiosity) and unfulfilled needs [4, 17, 18], as well as the desire to “be in a permanent state of an emotional high” or in love [3,4] or experiencing emotional tension: “People who are prone to polyamory are more into constant excitation and maintaining erotic arousal and their love life than the stability of the relationship.” [12]

Some of the experts pointed to social phenomena that explain the rising interest in polyamory, such as pressure to be happy or the wide access to contemporary mass culture messaging, which encourages us to seek happiness in our own way and off the beaten track. Such desires are deemed naive and harmful in the long run, exemplifying “the great sham of the contemporary world” [13]. Others explained people’s interest in polyamory with disappointment in monogamy and an inability to process the problems from previous relationships [7].

One therapist, having been asked to give a profile of polyamorous persons, said that they are people with very diverse experiences, personalities and libido levels, but that they are usually very mature and intelligent because they are the only ones who can shoulder the many challenges of an open relationship arrangement [20]. Another therapist described polyamorous people as “courageous enthusiasts of an amazing cultural experiment” [4].

#### Interpretative Analysis of the Profile of a Polyamorous Person

The experts had a clear tendency to create profiles of polyamorous people. As the quotations have shown, in most cases the profiles contained decidedly negative evaluations that point to unfavorable psychological traits, social correlates and problematic backgrounds. The experts suppose that choosing a polyamorous lifestyle is a form of escape from the responsibility and restrictions that naturally occur in good relationships. The source of this aversion to limitations is considered to be an aspect of the special personality of people who practice polyamory: narcissistic, egoistic and incapable of sacrificing for another person. Such strongly formulated assumptions about the pathological nature of polyamorous people may result from the clinical inclinations of some of the studied experts, therapists and scientists. Being used to explaining clinical diagnoses of what is unknown or deviating from the statistical norm, they reach for a stable repertoire of labels and theories, which, with an appropriate dose of creativity, can always explain the observed—and in their opinion undesirable—phenomenon.

The original assumption on which the subsequent search for the pathological traits of a polyamorous person is based is the impossibility of real intimacy and involvement in a multi-person relationship. So if a person decides to have such a relationship, the main motive for their actions is, as the experts suspect, the desire to escape from intimacy and involvement. The experts then try to explain this fear of closeness in people who choose polyamory by “an insecure attachment style” or by particular psychopathology (e.g. narcissism). The fact that some people decide to choose a polyamorous way of life without suffering is explained by the influence of culture—the current fashion for a “good-sounding” polyamory label allows them not to face their own deficits. On the other hand, this growing social consent to create non-standard relationships in the opinion of many of the experts deprives people of the chance to create an authentic, intimate relationship (which, in their opinion, can only be a monogamous one). According to some psychologists whose statements we have studied, if a polyamorous individual notices his emotional and personal shortcomings and works through them in the process of therapy, he could return to the socially and psychologically correct path that leads to the creation of a lasting monogamous relationship.

The experts devote a lot of attention to sexual matters, even if in the opening statement journalists define polyamory as a desire for establishing emotional bonds with multiple persons. Polyamory is often linked to diverse sexual adventures and to cheating. This suggests to the audience that these phenomena are similar in some crucial way. As a result, the audience is under the impression that polyamory is predominantly about having casual sex with many people.

The assumed openness of polyamorous people to sexual sensations is not assessed by the experts as positive or neutral. On the contrary, it is usually associated with cheating and sex addiction. It seems that emphasizing high sexuality as a source of interest in polyamory is aimed at inducing social antipathy. It refers to most people’s beliefs that an excess in the sexual sphere is inherently bad.

In addition to exploiting popular beliefs about sex in society, the analyzed experts also try to influence public opinion by using specific words to describe polyamorous relationships. In many statements, negatively charged terms dominate such as debauchery, promiscuity, betrayal, disloyalty and dishonesty. The experts also try to arouse aversion to polyamorous persons by emphasizing their alleged socioeconomic origin. The depiction of polyamorists as a leisure class permeated with new ideology is both mocking and contemptuous.

As in the case of the inconsistency between the naturalness and ease of monogamous relationships and the necessity of constant painstaking work on their condition, the experts’ opinions of the characteristics of polyamorous individuals also abound in contradictions. On the one hand, the choice of a polyamorous lifestyle is seen as an indicator of maturity because it is an expression of the partners’ consent to search for the most suitable relationship model. On the other hand, it is described as a weakness—an effect of succumbing to pressure exerted by the partner. The experts rarely notice that it is possible for two or more partners to decide on a polyamorous lifestyle in an equal, symmetrical and mutually beneficial way.

The experts draw up a simple scheme to show the motives behind starting to search for a relationship that is not monogamous. Usually, the initiative is attributed to a man who is selfish, hungry for thrills, and who has already found a candidate for a new partner. A new form of relationship is then forced upon a woman (usually presented as a weaker and more dependent person) who has too much to lose to resist. The result of such an “apparent agreement” must, according to the experts, be harm to either side.

### The Consequences

#### Insecurity or Relationship Instability

The most frequently mentioned feature of a polyamorous relationship is its instability and unpredictability, which lead to a sense of insecurity of the partners engaged in it [3,4,5,7,8,10,13,15]. As one of the experts puts it, “Another bane of a polyamorous person’s existence is insecurity” [4].

The supposed instability of a polyamorous relationship is linked to the assumption that polyamorists only seek high affective quality in a relationship, and once it fades the relationship falls apart [3]. The presumed instability is also explained by a stronger threat of conflicts, which multiply in a group bigger than two people [5], by difficulties reaching compromise and trouble communicating [13] and by conflicts of interest and dependencies [12]. For all these reasons, according to the majority of the studied experts, “polyamorists’ relationships can’t be stable” [3]. In contrast, a monogamous relationship is associated with boundaries and stability: “I wouldn’t want to be “the other one” for my man. I also wouldn’t want to have “another one.” Having boundaries provides a sense of security” [13].

Some of the experts presume that polyamorous relationships end when true love appears: “then the whole polyamorous theory falls apart because we want to give more prominence to this one relationship” [15]. An important life experience, for example the death of a parent, can test the authenticity of commitment and demarcate the border of the polyamorous network [15].

#### Jealousy

Another crucial category brought forth by the experts is jealousy. Not experiencing jealousy is for some of the experts evidence of a lack of love, commitment or desire to be together [12]: “If—as polyamorous people declare—it’s not at all there, it begs the question of how strong the commitment is” [7].

Jealousy is also presented as “an adaptive emotion which serves the protection of monogamy” [7]. This emotion, as the experts try to prove, guarantees “continuation of the species and survival” [8], is necessary “in order to be able to raise the human child, which is dependent for an unbelievably long time” [12] and is a consequence of “the biological resistance to investing in somebody else’s offspring” [1]. The experts also assume, as has already been mentioned, that humans are naturally monogamous creatures [12,20].

Because of the naturalness of jealousy, most of the experts believe that polyamorous persons have not really coped with jealousy in a constructive way, but are in denial about it [4,7,8,19]. It is believed that this defense mechanism may have profound negative effects on their mental health [7,8,19]:

Denial is unhealthy and carries repercussions in the area of emotions and even health. In a few years, every emotion we’re in denial about will come forth as depression, neurosis, insomnia or addiction. [8]

Apart from psychological effects, the experts also mention the accumulation of tension in the body, migraines, and other somatic symptoms that are the result of pushing down jealousy [19].

Jealousy in the media narrative is a natural emotion which appears as a reaction to the fear of being abandoned by the partner [12].Creating and maintaining polyamorous relationships provide, the experts say, too many reasons for such anxiety:

I become jealous when I suspect I might be left out in the cold. That’s why I think that in the case of polyamorists the level of jealousy must paradoxically be much higher than in monogamous relationships because it’s not only a suspicion that an alternative lover may appear. They are already there, which means that I’m no longer enough (…) while in a relationship between two people the partner doesn’t always give us a reason to feel jealous, so in a multi-person network it [jealousy] is inscribed there by definition. The threat of cheating is not a suspicion here, it’s a fact. [12]

#### Excess of Emotion

The experts point out that polyamorous relationships are not in danger of boredom [12,20]. They are, however, in danger of the opposite: an excess of emotions, mainly negative ones, which we are often not ready for [13,15]. Because of this “the emotional cost of such an arrangement is tremendous” [4]. One psychologist also fears that this amount of emotion can be difficult to handle: “in such relationships, people may be experiencing a lot of emotions, more than in a two-person relationship, (…) but one needs to be able to process all of that emotion.” [20]

This expert also points out that few people are capable of handling so much emotion and frustration, and so polyamory will remain a niche choice [20].

For one of the experts, who sees polyamory mainly as sexual exploration, the emotions that accompany sex are threatening because they are unexpected: “It may also be that the sex will bring forth some emotions in the other person which we didn’t expect. They can grow attached, start to commit, feel jealous, anxious, suffer. Sex is not pure fun.” [15]

#### Emptiness and Lack of Meaning of Life

The aforementioned lack of boredom and excess of emotion in polyamory is a temporary experience in some of the experts’ opinions. Later comes inevitable emptiness, or even “a loss of the sense of meaning of life” [8]. It is the result of higher tolerance for stimuli (in love and in sex “appetite comes with eating” [1]), which in turn leads to distraction and the inability to pause and focus on one thing [8]. In the end, such people are emotionally burnt out. After a longer time, polyamorous people are therefore “disheartened with themselves and life in general—they claim that there’s nothing more awaiting them” [8], and sex in their relationships “may strengthen the sense of ‘the shadow trail’ and the feeling that ‘nothing makes sense’” [15]. This emptiness is “something akin to professional burnout” [1], which cannot be easily overcome.

#### Diffusion of Responsibility and Social Indolence

Some of the psychologists assume that the presence of more than two partners must entail two negative effects described by social psychology: the diffusion of responsibility [9,13] and social indolence [9]. The former brings “a certain mental comfort,” although it has negative consequences for everyone involved [9]. The latter “is based on the fact that the more people are involved in collective action, the lower the average engagement of each of them in the results achieved. In other words, in collective action, some parasitize others” [9].

#### Influence on Children

The subject of the influence of the polyamory of parents on their children was referenced in a few of the interviews [1,4,13,15,19]. In some of them it was assumed that polyamorous relationships have to be harmful for children:

A known Polish actor. Raised by a mother from Sweden, liberal in the Swedish way, maintaining a few parallel relationships. Today he’s past forty and so far he’s had 500 women. He’s a sexaholic, he maintains a few relationships at the same time. Not one of them can be called “a stable one” [4].

In this example, a child raised by a polyamorous mother experiences envy of the mother’s time, unintended competition with the mother’s partners, a feeling of deficit in terms of attention, time and affection [4].

For some of the experts, the wellbeing of children depends on the parents’ preoccupation with lovers:

Children brought up in hippie families did very well. It can be similar here. (…) While a traditional family spends the weekend together, a woman who has, for example, two men, will divide her spare time between them, which can have an impact on her relationship with her children. It’s the same with men. [1]

Some of the experts would admit that in non-Western societies a (stable) polyamorous relationship might have some positive effect on children. But in our Western world, where monogamy is prevalent and highly valued, a child must feel insecure:

If this arrangement took place on a desert island and was stable, then those kids could be the happiest kids in the world because they’d have security and stability, which is the most important thing, they wouldn’t experience comparison and social judgement. In our environment (…) a kid like that can feel excluded and, as a result, not necessarily secure in this relationship. [5]

There are a few voices, however, which point out that in certain parts of the world the raising of children by the community or by a few adults does not have a negative impact on their development [15,19], and the parents’ lifestyle model facilitates parenting as no extra outside help is necessary to take care of the children [19].

#### More Difficult Life

Quite often these experts point out that polyamory, interpreted as a lifestyle, is difficult and requires a lot of effort from the people engaged in it. This effort is related to the lack of accepted patterns and ready-made behavioral scripts that monogamous relationships have at their disposal. The absence of norms forces people who practice polyamory to “create for themselves completely new definitions of love, responsibility, loyalty and the like because they just don’t fit into the existing definitions [12].

The experts describe polyamory as a kind of relationship which is very challenging, less orderly and less predictable than monogamy [9]. Polyamory requires not only the processing of an excess of emotions but also the ability to cope with them [20]. One of the experts assumes that in a multi-person relationship “issues of dependencies and interests have to be a real nest of problems” [12]. For some of the studied experts, also issues of planning, managing time, space and the availability of the other person are much more difficult in polyamory than in monogamy, all of which testifies to the advantage of the latter over the former: “There’s this saying: the real problem is to make two people want the same thing at the same time, and then if it’s not two but a dozen, we start to have problems, who with whom, when, how” [20].

Additionally, polyamory requires attention, time, energy in order to meet its requirements—some are clearly skeptical of the possibility of having these kinds of resources [9]. Others concede that with an adequate amount of effort, it is doable. One of the experts compares polyamory to cultivating more than one garden. All of them need weeding, watering, looking after. The essence of perfect polyamory is devoting attention, time and care to every partner. [4]

Sometimes, however, the experts claim just the opposite: that polyamory is an easy type of relationship that does not require effort [1,13,15] or even that it is a simplified behavioral script—“a ready-made pattern” [17].

#### Interpretative Analysis of the Assumed Consequences of a Polyamorous Lifestyle

As we have tried to show in previous parts of the analysis, the views of the experts on polyamory are largely shaped by the relationship models they promote and their beliefs concerning the psychological properties of people who seek non-traditional forms of life. The experts also try to justify their beliefs and negative attitudes towards polyamory by pointing out the adverse consequences that, in their opinion, this form of relationship entails.

A large number of the studied experts claim that polyamorous relationships are, by their very nature, very unstable. They do not cite any empirical data to support this thesis but try to justify it by using common-sense beliefs. They point out that a larger number of people in an intimate relationship must necessarily result in a larger number of needs at stake—the necessity of permanent searching for compromise and constant negotiations in search of a model of life that is acceptable to all. In this context, one feature of monogamous relationships is recalled once again: they are based on clear and unchanging rules which give a sense of predictability and security. Polyamorous relationships, on the other hand, give rise to socially unfavorable phenomena such as social idleness and diffusion of responsibility. Some of the experts predict that such relationships will sooner or later fail.

The experts indicate several reasons for this inevitable failure. One of them is the circumstance in which one of the persons in a polyamorous relationship finds his or her “true love” (based on the previously discussed assumption that this kind of love cannot be experienced in relation to more than one person and is a simple consequence of the adoption of the myth of “two halves”). For other people, difficult circumstances in life will disprove the declarations of equal commitment of all the people forming the relationship. The experts imagine crisis situations in which only one of the many partners will provide enough understanding, help and support. This will be the one who is truly devoted and loving and with whom the person who is experiencing a crisis will later want to bond exclusively. Ultimately, therefore, according to the experts every polyamorous relationship must fail or be transformed into monogamy.

The experts also do not believe that the problem of jealousy in close relationships can be resolved constructively as this emotion seems natural and evolutionarily adaptive to them. Polyamorists who claim to have dealt with jealousy either lie or—in the name of the desire to remain in an unusual relationship—suppress their healthy instincts. Such denial of one’s own emotions must, in the opinion of the experts, have serious negative somatic and psychological consequences.

It is worth noticing that polyamory is defined by many of the experts as cheating on and excluding a partner. It does not seem relevant to them that when new people join the relationship, none of the others leave. The sheer fact of being interested in a new person is interpreted as abandonment of the current partner(s).

The experts are also convinced that polyamorous relationships generate an excess of emotions which are difficult to deal with and which are very exhausting in the long run. Many of them are associated with the excitement of sexual intercourse. However, according to some of the studied experts, this kind of physical closeness also has a darker side: it can awaken dormant desires, emotions and reactions. Sex reveals layers of emotion in people that they may not have known before. It is dangerous then to play with sex.

The experts assume that people who create polyamorous relationships are guided by the need to constantly experience strong emotions and with time this leads to habituation and the consequent necessity to experience even stronger sensations. Ultimately, it brings not fulfilment but extreme exhaustion. The studied experts also believe that desire they find in polyamorous people—to seek newer and newer experiences results from the feeling of existential emptiness. The one who seeks must feel a lack. A fulfilled person is content with little.

In addition to the negative consequences of polyamory for partners, some of the experts fear the harmful effects on children growing up in such relationships. Sometimes the fear is expressed that the parent’s way of life will have a direct negative impact on the child, who will later take on the parental pattern of casual, uncommitted sexual contacts. More often the experts point to an increased risk of the child’s needs being neglected, but this can be prevented. If, despite their emotional involvement in numerous partners, the parent has sufficient time and attention for the child, the child can get out of it unscathed. It seems that this way of thinking is based on two assumptions: the vision of an ideal childhood (when a lot of attention is paid to the child by the parents) and the belief that a polyamorous relationship is engaging and exhausting for its participants.

Some of the experts can imagine a happy childhood in a multi-partner family. In their opinion, we have anthropological evidence of this possibility. However, even those who are familiar with these accounts point to a different cultural context. In Western societies, where monogamy is the dominant family model, children brought up in “non-standard” families are exposed to stigmatization and social exclusion. It is worth noting that the experts do not feel the need to change social attitudes towards new forms of relationships that would make it possible to avoid the negative consequences of misunderstanding and intolerance. In their statements they instead contribute to the perpetuation of the status quo and the attribution of possible blame for the consequences of risky life choices to parents.

Another consequence of maintaining a multi-partner relationship is, according to the experts, the fact that it is excessively complicated. This results from the need to create one’s own norms and rules, to undertake constant work on difficult emotions that may arise, to agree on the needs and interests of many people, and to devote more time and attention to the relationship than is the case in monogamy.

At the same time, the experts seem to miss the fact that when identifying the qualities of polyamorous relationships, they often contradict themselves. The same relationship, which is meant to be an escape from commitment (which is seen as a personality defect), requires a great deal of commitment (which is seen as a relationship defect). The same relationship, which is supposed to be devoid of rules by its very nature, can be presented as difficult because of the need to create one’s own rules. People who, according to the experts, are unable to devote time and attention to their partner choose relationships in which they have to devote more time and attention than would be the case in a monogamous relationship. Polyamorous relationships are presented by some as complicated and time and energy consuming, but by others they are described as an effortless, ready-made template for the emotionally lazy. However, no matter what features are attributed to polyamorous relationships, the emerging result is almost always to deter people from entering them.

## Discussion

The analysis of Polish media discourse on polyamory shows that most psychologists and sexologists perceive polyamory as incompatible with the principles of healthy love. They consider people who are polyamorous as devoid of the significant psychological resources necessary to establish good relationships. The polyamorous lifestyle is presented as riddled with risk. To sum up, on the basis of our sample we are inclined to think that the mononormative social and moral order is often reinforced and justified in the public discourse by representatives of psychology and sexology. This is done in a way that gives an appearance of scientific credibility.

The experts express their opinions in a firm manner. If psychological theories appear in their statements, they are very general and not closely related to the topic of polyamory. The concepts most frequently referred to by the studied experts include Sternberg’s theory on intimacy, commitment and passion, or psychoanalytic approaches to object relations, attachment disorders, and defense mechanisms. The concepts are selectively interpreted to strengthen the superiority of social arrangements based on sexual and emotional exclusivity in a relationship.

The experts do not refer to any empirical research conducted on polyamorists. Their statements about human nature and relationships are only seemingly scientific. Quite freely quoted fragments of psychological theories serve the experts as a kind of smoke screen by which they try to conceal their private opinions and moral judgments concerning polyamory. These views are largely shaped by the mono-normative culture in which the experts live.

This strategy of employing psychological jargon to back up the negative moral assessment of people and relationships that are consensually nonmonogamous is not a new one. In fact, it was noticed and documented by other scholars several decades ago. Research carried out in the 1970s showed that people who enter CNM relationships were perceived by therapists as narcissistic, emotionally burnt out, acting out, impulsive, immature and irresponsible (Constantine et al., 1972). Knapp (1975) demonstrated that in the case of consensually nonmonogamous clients, as much as a third of therapists presumed personality disorders and neurotic tendencies, and a fifth assumed asocial personality. In the research conducted a decade later by Hymer and Rubin (1982), a quarter of therapists made ad hoc assumptions about people in open relationships experiencing fear of commitment and intimacy, and 7% assumed that the partners may have an identity problem.

Our own research on statements about polyamory made in media interviews by Polish experts confirms these findings. It demonstrates that the repertoire of negative evaluations and psychopathological labels used with a sub-group of CNM clients has not changed much with time. This could beg the question of whether these traits are attributed solely to persons who choose a nonmonogamous lifestyle or perhaps serve as a general go-to label toolkit. These labels serve to justify the therapists’ aversion and prejudice towards certain groups of people. Certain crucial similarities can definitely be identified between the descriptions of homosexual people made by psychoanalysts in the 1970s and the descriptions of people who choose nonmonogamous relationship models (cf. discussion regarding the genesis of homosexuality and homosexual people’s traits in Stoller et al. (1973)).

The results of newer studies also confirm the negative or partly unfavorable attitude of therapists towards polyamorous people. These relationships are usually perceived as based only on apparent consent—lacking a sense of security, stability, intimacy, closeness, or support from the partner (Grunt-Mejer & Łyś, 2019). There is no research that would directly show how the attitudes and opinions of therapists manifest themselves in the process of therapeutic work with people who are polyamourous. There are only reports on the opinions of therapy clients who live in polyamorous relationships (Henrich & Trawinski, 2016; Schechinger et al., 2018). These accounts allow the cautious hypothesis that the prejudices of therapists, even if they occur, do not necessarily have to be manifested in the course of therapy.

As we have shown, most experts, when they act as a social authority, choose a strategy that aims to perpetuate the traditional values and beliefs that the only proper form of intimate relationship is a monogamous relationship. It is worth considering why this happens.

The first hypothesis is based on the automatic antipathy that emerges from some of the experts’ emotionally charged statements and is directed to relationship models other than the monogamous one. These kinds of reactions can be deemed partly understandable if we take into account that polyamory is a double transgression against the commonly accepted norms in romantic relationships—norms that are probably also shared by the experts. Firstly, it means consent to maintaining sexual relations with more than one person; secondly, it allows romantic feelings towards a few people at the same time. In a mono-normative culture, each of those transgressions is automatically associated with cheating, which is a situation where one person suffers harm and the continuation of the relationship is in question. The analyzed experts’ aversion to polyamorous relationships and their tendency to formulate negative assessments of them can then be explained by the fact that they are seemingly similar to unfaithfulness. In other words, polyamory can evoke emotions such as betrayal, disgust, anger, or fear, which in turn can form the basis for the experts’ reluctant attitudes.

However, the clear differences between polyamory and betrayal demand justification of a negative attitude towards the former, other than the very fact that it arouses emotions. The social intuitionism model (Haidt, 2013) offers an explanation of the formation process of such a justification. According to this model, in a situation in which someone transgresses a social norm that is difficult to justify other than by referring to tradition, people automatically react in a negative way. Nevertheless, when they are forced to explain the reasons for their reaction, they tend to give ad hoc explanations which at least at first glance seem rational. The psychologists we analyzed offer such rationalizations. Some of them explicitly formulate unfavorable moral evaluations of polyamorous relationships. Others, often confronted by journalists with examples of polyamorous relationships where no one seems to be harmed, feel the need to more “scientifically” support their intuitive assessment. They then fall back on the familiar tools in the form of psychological labels and theories.

The other reason for these negative attitudes may stem from the form of professional training of sexologists and therapists. The most popular academic books on the subject of love and relationships are based on theories that describe the dynamics of monogamous relationships. Sexually and emotionally open relationships are either not described at all or are presented in the context of relational problems, such as the above examples from psychodynamic publications. Also, sexology studies in Poland focus primarily on clinical and developmental sexology (often based on older psychoanalytic theories which are presented in a way that suggests that true attachment can be to only one person). The current curricula of most university psychology and post-graduate sexology courses in Poland do not raise the topic of CNM relationships. The only academically discussed area of non-normative sexual choices is same-sex relationships, but these are also presented as a simple reflection of “ideal” heterosexual relationships (i.e. permanent and monogamous). A therapist wishing to gain knowledge about other non-normative sexual choices is forced to look for rare niche courses beyond the curricula of the largest psychological and sexual schools. This situation may translate into a lack of knowledge about polyamory among Polish psychotherapists and sexologists. Also, professional psychological and sexual associations in Poland do not offer any resources or statements aimed at raising awareness of relational diversity. Based on the positive results of the dissemination of knowledge on sexual orientation and affirmative approaches in LGB therapy, such official statements could influence attitudes towards polyamory among mental health professionals.

## Appendix 1

Source list in chronological order:

1. Poliamoria. Jeden facet to za mało. Wywiad z Arkadiuszem Bilejczykiem. [Polyamory. One guy is not enough. An interview with Arkadiusz Bilejczyk.] [video]. (2012, May 04). *Wirtualna Polska Kobieta*. Retrieved from https://kobieta.wp.pl/poliamoria-jeden-facet-to-za-malo-5982660874965633a?ticaid=11358d.
2. Chilewicz, P. (2012, July 10). Poliamoria. Wielu partnerów w związku = więcej miłości? Czy wręcz przeciwnie? Wywiad z Agatą Loewe. [Polyamory. Many relationship partners = more love? Or to the contrary? An interview with Agata Loewe.] *Na Temat*. Retrieved from http://natemat.pl/22633,poliamoria-wielu-partnerow-w-zwiazku-wiecej-milosci-czy-wrecz-przeciwnie.
3. Domańska, A. (2012, July 20). Poliamoria: Wszystko w rodzinie. Wywiad z Małgorzatą Zaryczną. [Polyamory. All in the family. An interview with Małgorzata Zaryczna.]. *Zwierciadło*. Retrieved from http://zwierciadlo.pl/seks/erotyka/poliamoria-wszystko-w-rodzinie.
4. Hajn, U. (2012, October 07). Poliamoria - sposób na udany związek czy ucieczka od wygasających namiętności? Wywiad z Andrzejem Gryżewskim, Grażyną Czubińską, Agatą Loewe i Adrianną Kłos. [Polyamory - a recipe for a successful relationship or an escape from fading passion? An interview with Andrzej Gryżewski, Grażyna Czubińska, Agata Loewe and Adrianna Kłos.] *Gazeta.pl Kobieta*. Retrieved fromhttp://kobieta.gazeta.pl/kobieta/1,107880,12606129,poliamoria-sposob-na-udany-zwiazek-czy-ucieczka-od-wygasajacych.html.
5. Czy poliamoria to sposób na udany związek? Wywiad z Danielem Cysarzem [Is polyamory a recipe for a successful relationship? An interview with Daniel Cysarz] [video]. (2012, November 27). *Wysokie Obcasy.pl*. Retrieved from http://kobieta.gazeta.pl/kobieta/12,88991,12932708.html?bo=1.
6. Niedźwiecka, M. (2012, December 06). Agata Loewe: Poliamorycy są wśród nas. Wywiad z Agatą Loewe. [Agata Loewe: Poliamorists are among us. An interview with Agata Loewe.] *Zwierciadło.pl*. Retrieved from http://zwierciadlo.pl/seks/erotyka/agata-loewe-poliamorycy-sa-wsrod-nas.
7. Zuchora, A. (2012, July 09). Poliamoria: Miłość w sieci. [Polyamory. Network love.] *Newsweek Polska*. Retrieved from http://polska.newsweek.pl/poliamoria–milosc-w-sieci,105835,1,1.html.
8. Rozwadowska, A. (2013, July 14). Seksuolożka: Poliamoria jak jazda na karuzeli. To niebezpieczny trend. Wywiad z Violettą Nowacką. [Sexologist: Polyamory is like riding a rollercoaster. It’s a dangerous trend. An interview with Violetta Nowacka.] *Głos Wielkopolski*. Retrieved from http://www.gloswielkopolski.pl/artykul/944463,seksuolozka-poliamoria-jak-jazda-na-karuzeli-to-niebezpieczny-trend,id,t.html?cookie=1.
9. Wodecka, D. (2014, January 31). Poliamoria to bzdura. Wywiad z Wiesławem Łukaszewskim. [Polyamory is nonsense. An interview with Wiesław Łukaszewski.] *Wyborcza, Magazyn Świąteczny*. Retrieved from http://wyborcza.pl/magazyn/1,124059,15366052,Poliamoria_to_bzdura.html.
10. Wesołowski, M. (2014, February 05). Poli boli. [Poly hurts.] *Wyborcza Duży Format*. Retrieved from http://wyborcza.pl/duzyformat/1,127290,15401794,Poli_boli.html.
11. Poliamoria - różne odcienie miłości. Wywiad z Zbigniewem Lew- Starowicz, Hanną Bakuła, Danielem Jabłońskim, Katarzyną Michalczak, Agnieszką Weseli i anonimową kobietą. [Polyamory - various shades of love. An interview with Zbigniew Lew- Starowicz, Hanna Bakuła, Daniel Jabłoński, Katarzyna Michalczak, Agnieszka Weseli and an anonymous woman. (2014, February 11). *TVP.pl*. Retrieved from http://pytanienasniadanie.tvp.pl/13963620/poliamoria-rozne-odcienie-milosci.
12. Pietryga, E. (2014, March 22). Poli tury. Wywiad z Bogdanem Wojciszke. [Poly turns. An interview with Bogdan Wojciszke.] *Rzeczpospolita*. Retrieved from http://www.rp.pl/artykul/1096005-Poli-tury.html.
13. Kisiel, A. & Gajda, D. (2014, March 28). Poliamoria - kiedy kocha się podwójnie. Wywiad z Joanną Drosio-Czaplińska i Danielem Cysarzem. [Polyamory - when you double love. An interview with Joanna Drosio-Czaplińska and Daniel Cysarz.] *Portal Onet*. Retrieved from http://kobieta.onet.pl/zdrowie/zycie-intymne/poliamoria-kiedy-kocha-sie-podwojnie/cmlps.
14. Pawłowicz, B. (2014, July 30). Na ratunek miłości: Mono czy poli? Ty decydujesz. Wywiad z Wojciechem Eichelberger. [Save the love: Mono or poly? You decide. An interview with Wojciech Eichelberger.] *Zwierciadło*. Retrieved from http://zwierciadlo.pl/2014/seks/na-ratunek-milosci-mono-czy-poli-ty-decydujesz.
15. Reiter, P. (2015, October 10). Kilka srok za ogon. Wywiad z Alicją Długołęcką. [A few birds with one stone. An interview with Alicja Długołęcka.] *Wysokie Obcasy*, *237*, 24–24. Retrieved from http://www.wysokieobcasy.pl/wysokie-obcasy/1,96950,18986226,seks-kilka-srok-za-ogon.html.
16. Romanowska, K. (2016, February 18). Ten trzeci. Ona, on i kochanek. Wywiad z Andrzejem Gryżewskim. [The third one. Her, him and a lover. An interview with Andrzej Gryżewski.] *Wysokie Obcasy*, *3*. Retrieved from http://www.wysokieobcasy.pl/wysokie-obcasy/7,152731,23164920,odkad-mam-kochanka-w-moim-malzenstwie-uklada-sie-lepiej-jestem.html.
17. Jucewicz, A. (2017, July 20). Gotowce, seks i my. Wywiad z Zofia Milska-Wrzosińska. [Ready-made patterns, sex and us. An interview with Zofia Milska-Wrzosińska.] *Wysokie Obcasy*, *8*. Retrieved from http://www.archiwum.wyborcza.pl/Archiwum/1,0,8273846,20170720RP-QOB,Gotowce_seks_i_my,.html.
18. Kuszewska, M. (2018, April 03). Razem we troje. Wywiad z Piotrem Gumiennym. [The three of us together. An interview with Piotr Gumienny.] *Esquire Polska*, *2*, 30–30.
19. Jamrozek, J. (2018, August 19). Poliamoria - kiedy kocha się wielu. Wywiad z Andrzejem Gryżewskim. [Polyamory - when you love many. An interview with Andrzej Gryżewski.] [radio broadcast]. *Czwórka PR*. Retrieved from https://www.polskieradio.pl/10/4023/Artykul/2191136,Poliamoria-kiedy-kocha-sie-wielu.
20. Darmetko, G. (2014, January 12). Poliamoria. W takich związkach ludziom nie grozi nuda? Wywiad z Bartoszem Zalewskim [Polyamory. Is boredom not a threat in these relationships? An interview with Bartosz Zalewski.] [radio broadcast]. *Trójka PR*. Retrieved from https://www.polskieradio.pl/9/3031/Artykul/1022439,Poliamoria-W-takich-zwiazkach-ludziom-nie-grozi-nuda.
